# A Huge Chondrosarcoma of the Nasal Septum

**DOI:** 10.7759/cureus.27532

**Published:** 2022-07-31

**Authors:** Azliana Aziz, Mohamed A. Malik Mohamed, Mohammad Habibullah Khan

**Affiliations:** 1 Otorhinolaryngology, Head and Neck Surgery (ORL-HNS), Universiti Sains Malaysia School of Medical Sciences, Kota Bharu, MYS; 2 Otorhinolaryngology, South Infirmary-Victoria University Hospital, Cork, IRL

**Keywords:** nasal obstruction surgery, radiotherapy, endoscopic surgery, chondrosarcoma, nasal septum

## Abstract

Chondrosarcoma of the nasal septum is a rare malignancy. Its early diagnosis is difficult because of its non-specific sinonasal complaints. We report a case report of a 72-year-old woman who presented with progressive nasal obstruction and anosmia. Nasal endoscopy showed a nasal mass obstructing both nasal cavities, which was not separable from the nasal septum. Endoscopic biopsy confirmed chondrosarcoma. The tumour was operated on with complete margins by an endoscopic approach. The clinical presentation of the disease, diagnosis and treatment of this case, and a review of the literature are discussed.

## Introduction

Sinonasal chondrosarcoma is a rare tumour that represents approximately 5%-10% of malignant primary bone tumours that occur in the head and neck region [1]. The nasal septum is a rare site of origin for sinonasal chondrosarcoma and accounts for about 0.1% of the sinonasal region [2]. Chondrosarcoma is a malignant tumour with tendencies to arise from pure hyaline cartilage differentiation and may occur from chondrocytes, embryonal rests or mesenchymal cells that undergo multidirectional differentiation to form either cartilaginous or osseous [3] elements. It can arise from either bone or soft tissue [4]. The site of predilection for the head and neck chondrosarcoma includes ethmoid sinus, maxilla, nasal septum, hard palate, nasopharynx, and alar cartilage [5]. They rarely extend into the cranial or intracranial areas unless there is a recurrence [6] of the disease.

## Case presentation

A very healthy and fit, 72-year-old lady was referred to an Otorhinolaryngology outpatient clinic with complaints of a slowly progressive history of nasal obstruction and anosmia for a few weeks. There was no history of vision loss, diplopia, proptosis, facial pain, facial asymmetry, weight loss or epistaxis. Intra-oral and ear examinations were normal.

**Figure 1:**
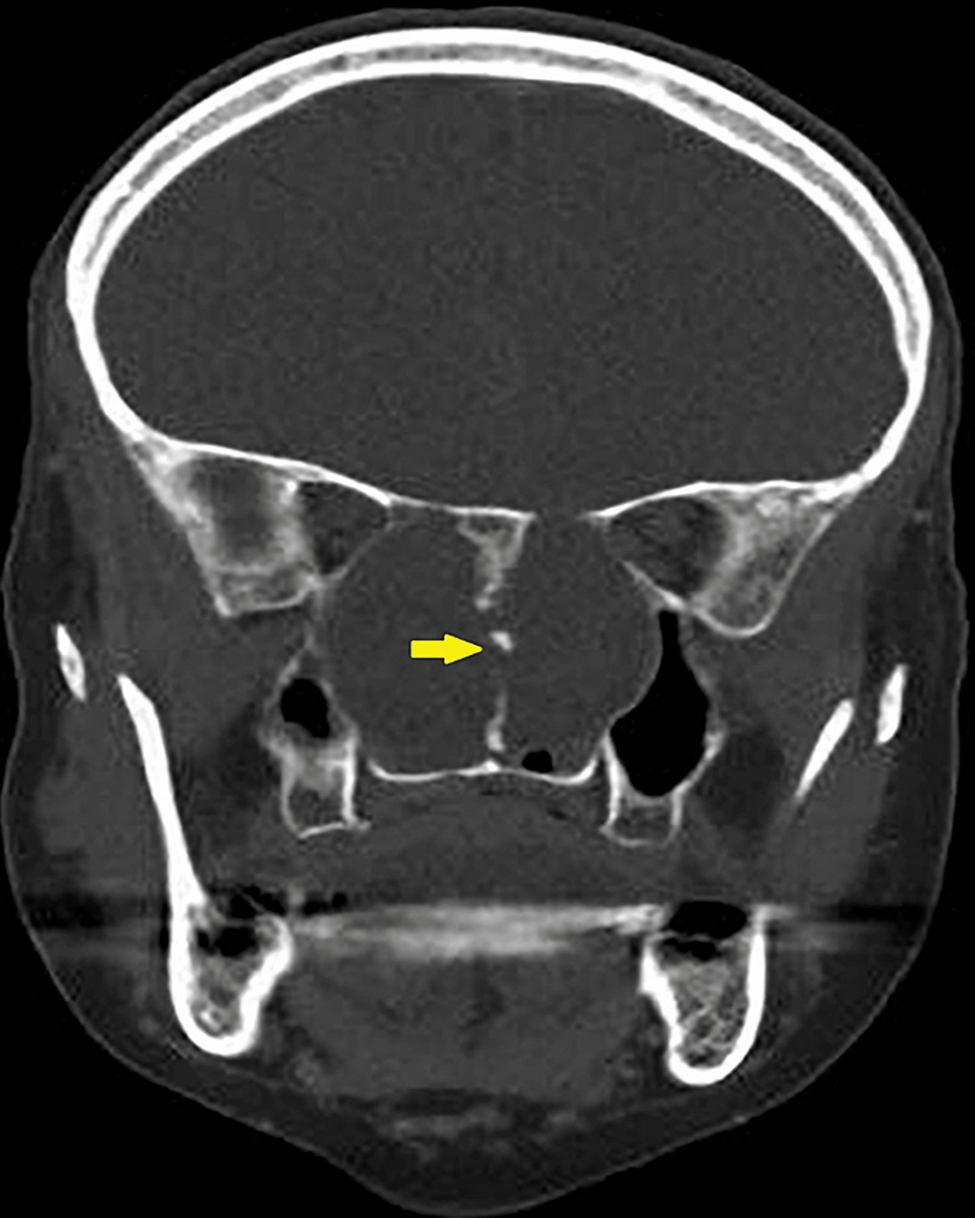
CT scan (coronal view) showing the soft tissue mass occupying the sinonasal cavity with the erosion of the septum (yellow arrow)

**Figure 2:**
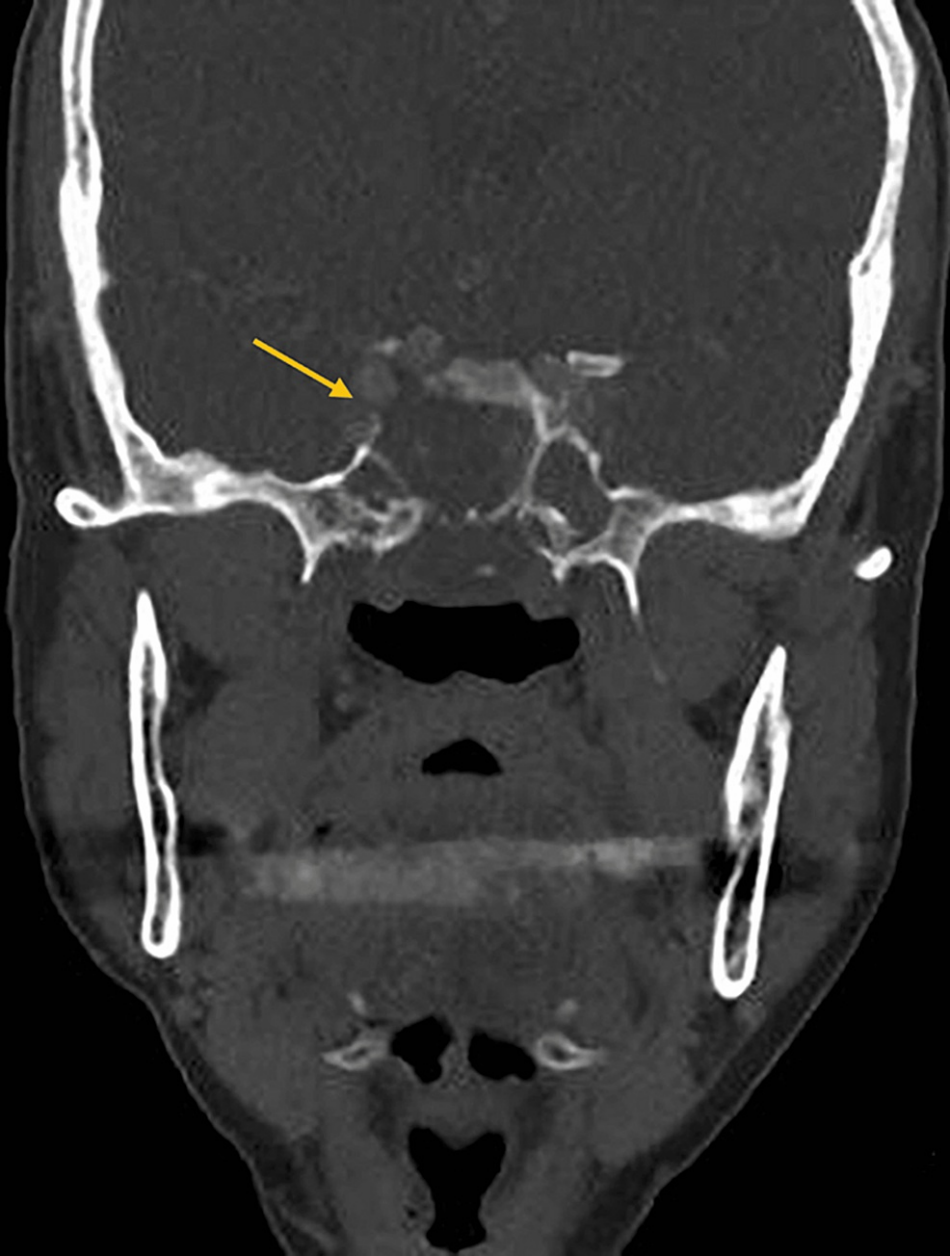
CT scan (coronal view) showing the tissue mass occupying the sinonasal cavity with the erosion of the sphenoid and clivus (yellow arrow)

The nasoendoscopy examination revealed a large central nasal mass occupying the nasal cavity. Both computed tomography (CT) and magnetic resonance imaging (MRI) was performed to fully evaluate the extension of the disease as well as the bone and soft-tissue characteristics of the lesion. CT scan of the sinuses showed a well-circumscribed sinonasal mass in the nasopharynx with the epicentre at the posterior nasal septum and expanding laterally that causing bowing of the medial nasal walls (Figure 1), eroding the sphenoid and clivus and exposing the right internal carotid artery (ICA) (Figure 2).

**Figure 3:**
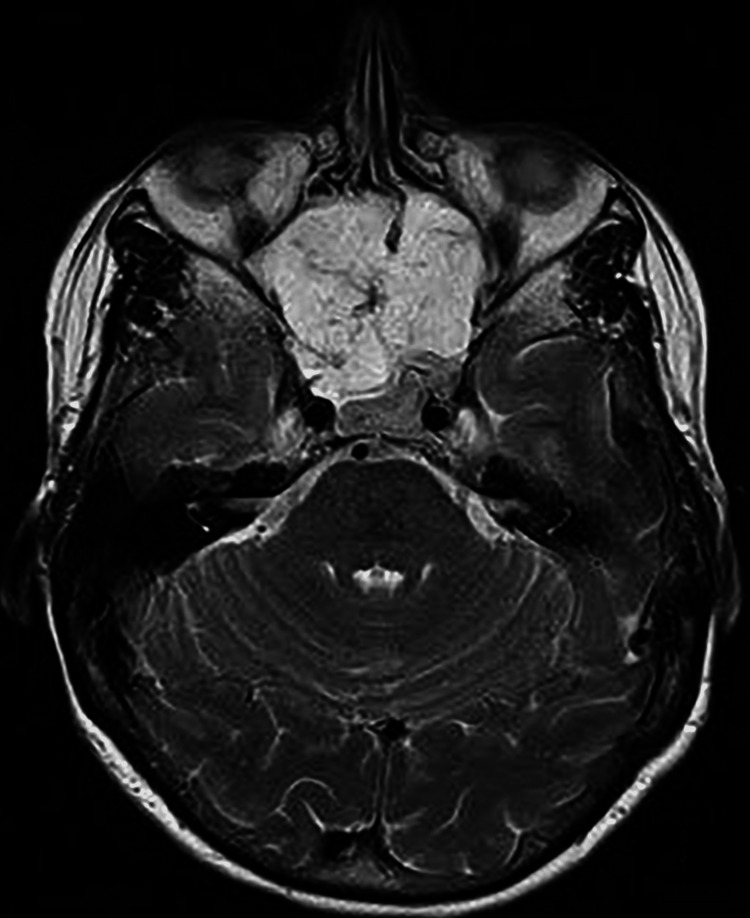
The axial view of MRI (T2 weighted image) showed a lesion abutting sphenoid cavernous sinuses posteriorly

**Figure 4:**
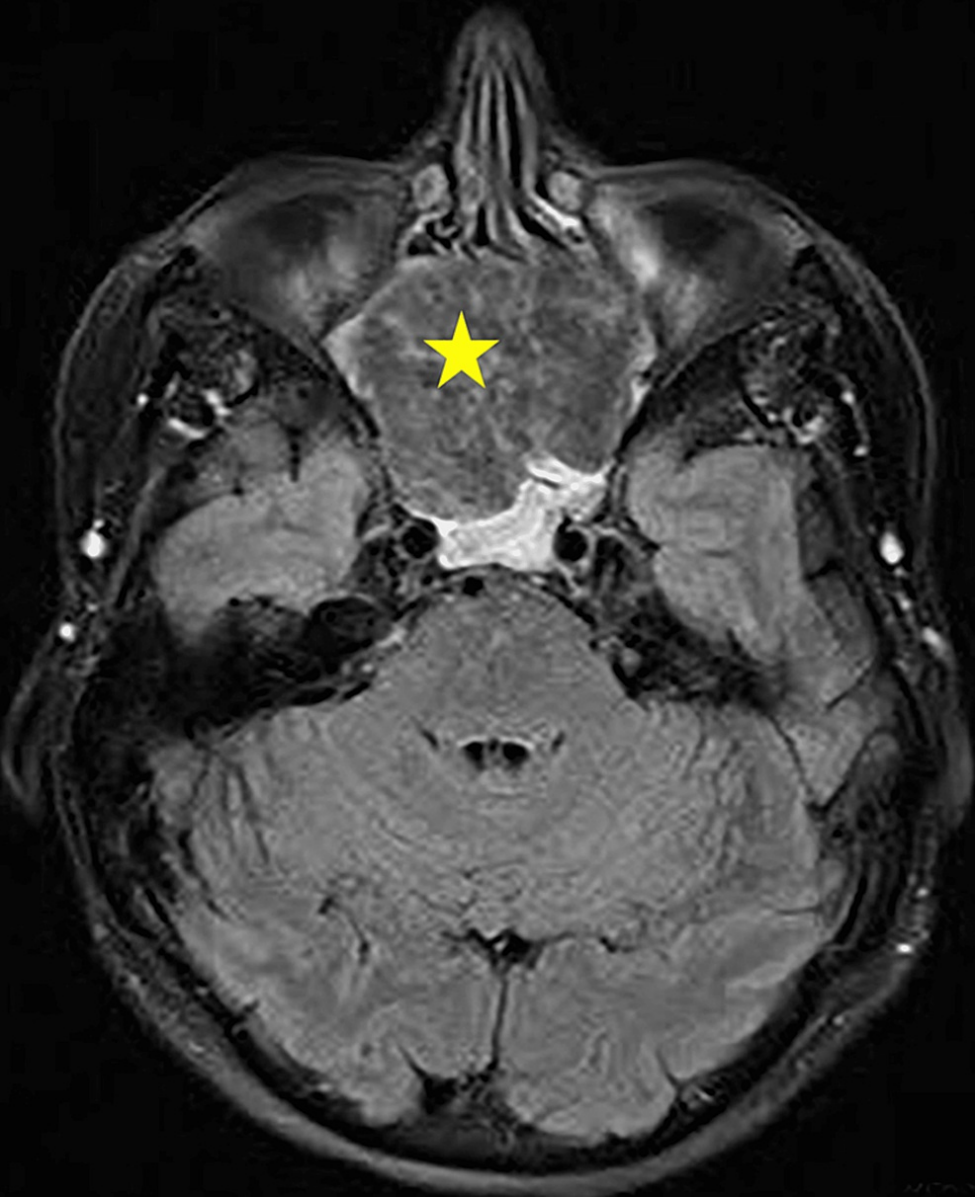
The axial view of MRI (T1 fat-suppressed image) showed a lesion (yellow star) abutting sphenoid cavernous sinuses posteriorly

**Figure 5:**
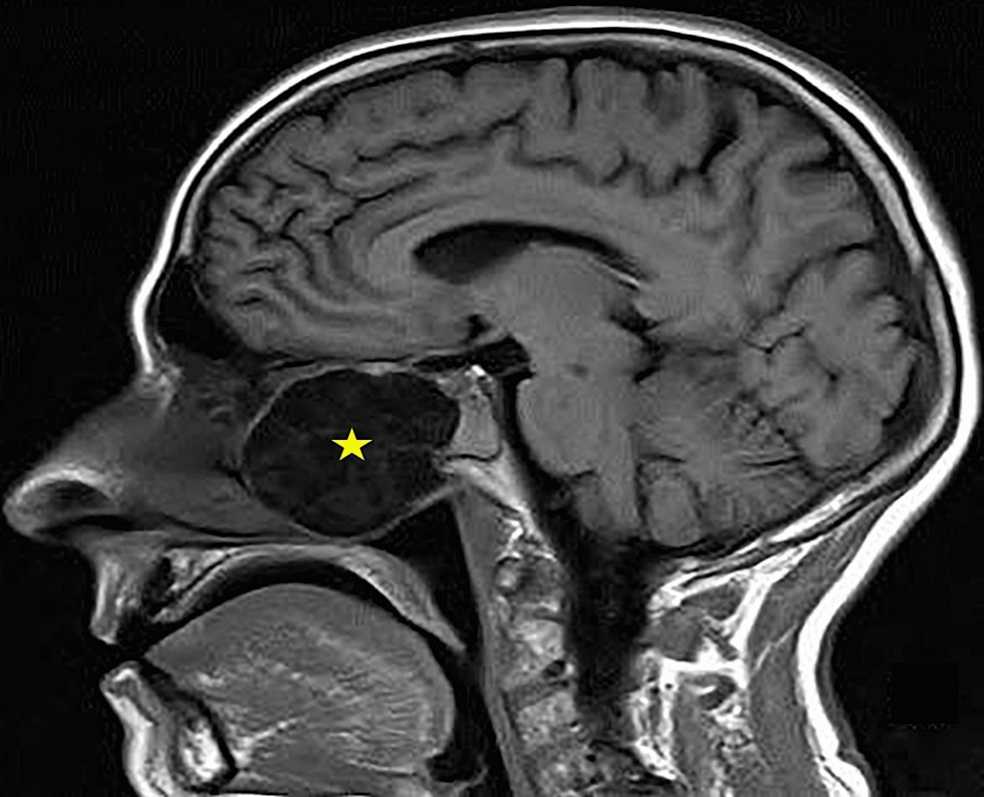
Sagittal view of the soft tissue mass MRI (yellow star) occupying the nasal cavity

**Figure 6:**
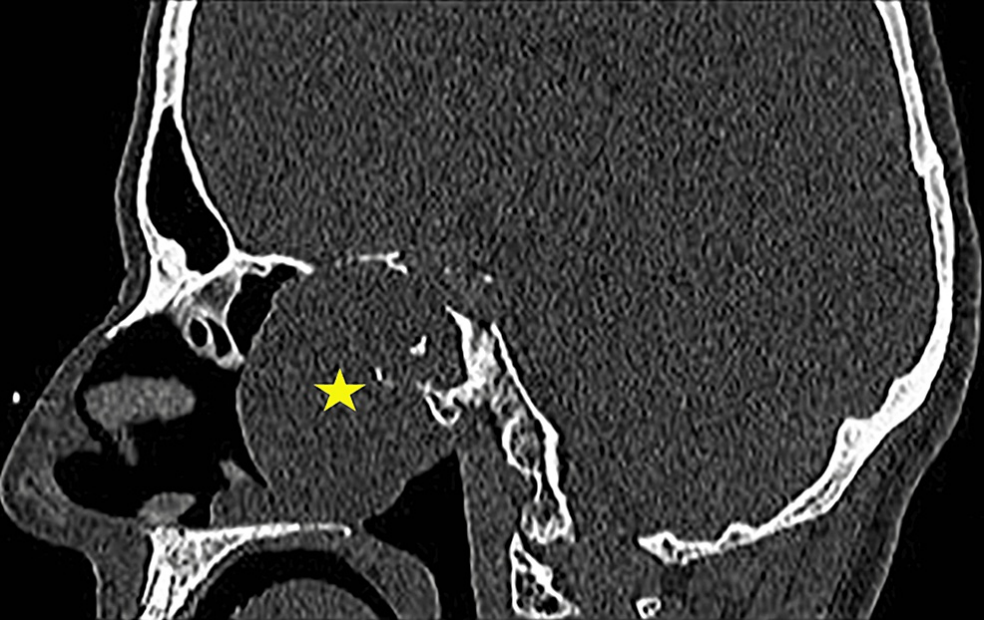
Sagittal view of mass in CT scan (yellow star) with the erosion of the skull base involving the sphenoid and clivus

The MRI showed a detailed definition of the nature, extent and size of the mass. It showed an eroded and deformed nasal septum with the lesion abutting sphenoid cavernous sinuses (Figures 3, 4) posteriorly. There was also a mild narrowing of the superior orbital fissure bilaterally, abutting the optic nerves bilaterally without definite involvement. Figures 5, 6 demonstrated the sagittal view of the lesion in the CT and MRI scan. There was no neck adenopathy or any distant metastasis noted.

 

Endoscopic biopsies were performed and the histopathology examination confirmed grade 2 chondrosarcoma. This case was discussed by our multidisciplinary team, the decision was taken to remove the tumour by an endoscopic approach under general anaesthesia. The patient underwent endoscopic endonasal transsphenoidal surgery, a combined case with a neurosurgical team. 

We used a Stealth station (Medtronic ENT Neuronavigation system). Intraoperatively, middle turbinates and lamina papyracea were identified and preserved. The whole of the posterior septum from the hard palate to the skull base was removed. Anterior skull base was drilled with minor cerebral spinal fluid (CSF) leak identified and sealed with factor Xa-like protein (Haemopatch) and DuraSeal. Sphenoid and clivus bone was drilled and right ICA was identified. There was no extension into the cavernous sinus was noted and the whole tumour was resected via an endoscopic approach.

**Figure 7:**
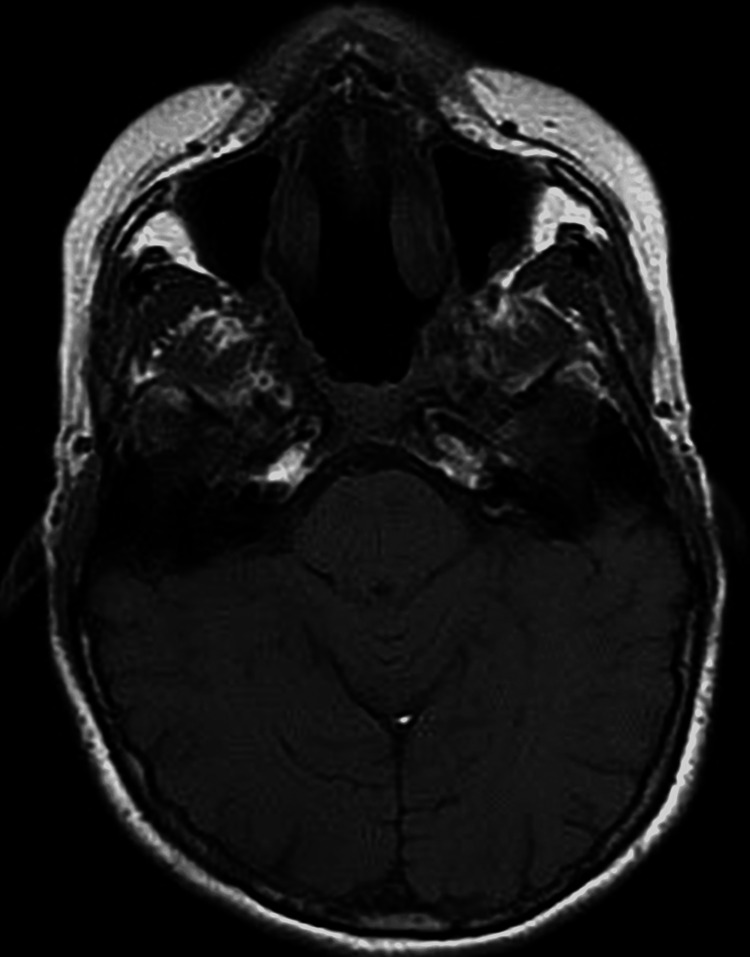
MRI (T1-weighted image) after four months of resection, which revealed complete removal of the tumour and preservation of the lateral structures

**Figure 8:**
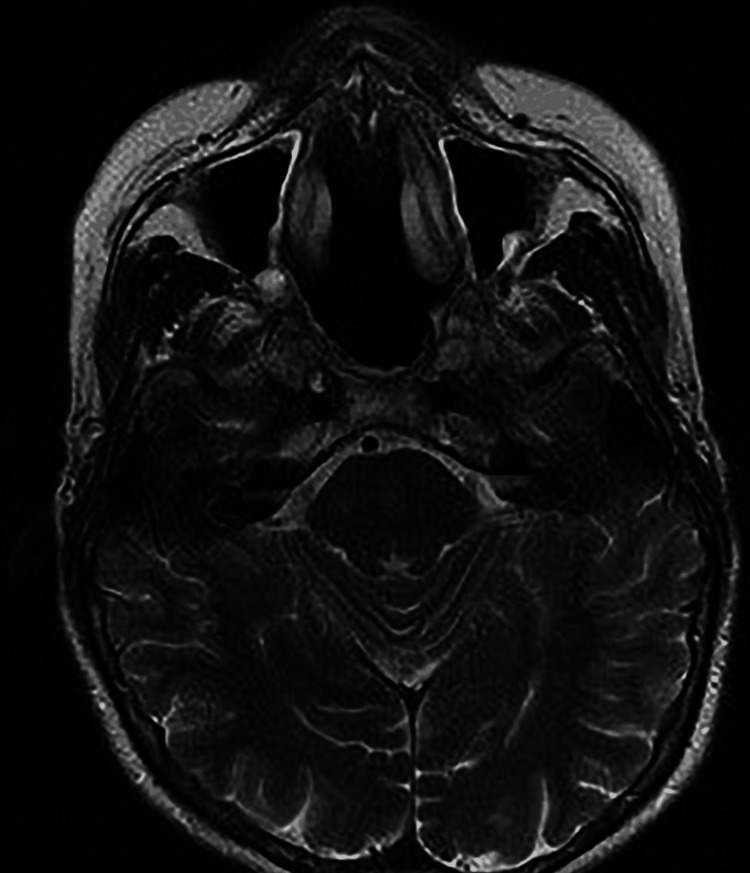
MRI (T2-weighted image) after four months of resection with preservation of the lateral structures and complete clearance of tumour

Post-operatively, the case was discussed in a multi-disciplinary team meeting for postoperative radiotherapy. The patient currently opted for endoscopic and radiologic surveillance for her follow-up. Repeat MRI images after four months of endoscopic resection of the tumour were carried out and demonstrated completed resection of the chondrosarcoma (Figures 7, 8).

**Figure 9:**
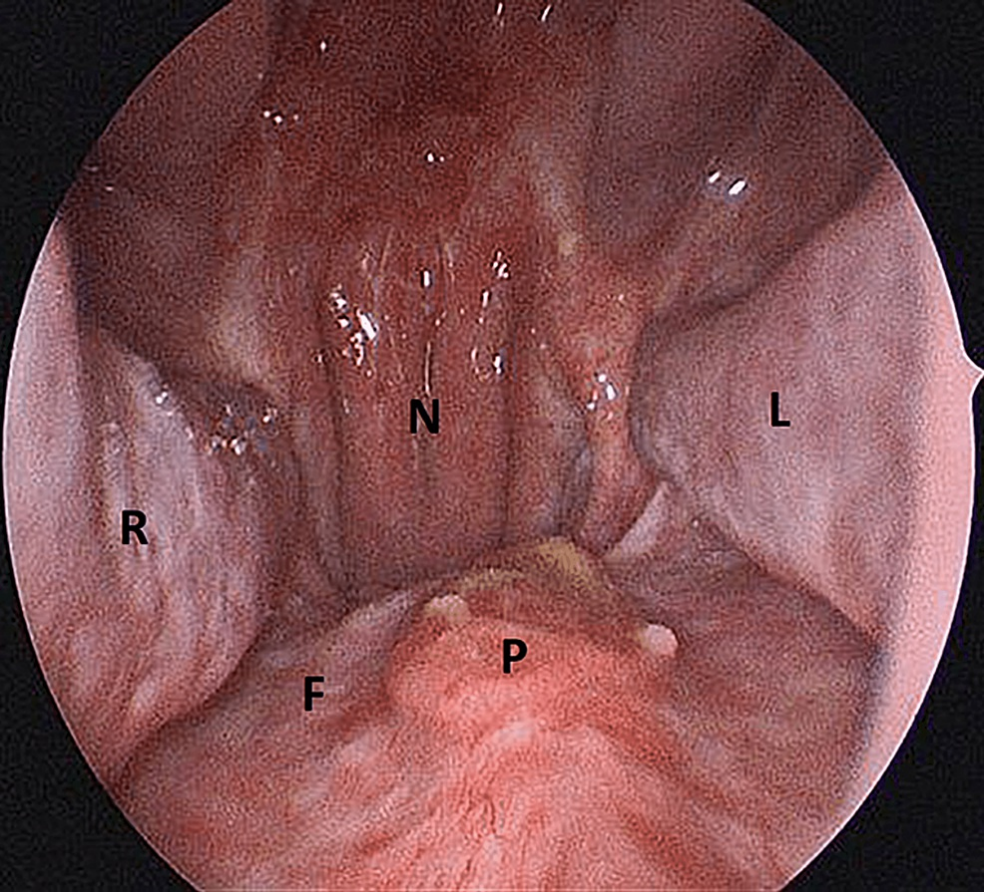
Endoscopic image taken during the time of biopsy after four months of initial resection R= Right inferior turbinate, L= Left inferior turbinate, F= Nasal floor, P= Palatine bone/hard palate, N= Nasopharynx

The patient underwent repeat biopsies and histological examination showed no disease recurrence. The endoscopic image during the time of biopsy after a few months of initial resection also revealed good healing after the operation (Figure 9).

## Discussion

Chondrosarcoma is a non-epithelial malignant tumour, which is a slow-growing tumour that usually involves the pelvis, ribs, long bones of the extremities, scapula, and the sternum. It affects men and age groups between 60 and 70 years old. Chondrosarcoma can be associated with conditions such as multiple hereditary exostoses, Ollier disease, Maffucci syndrome [7], earlier usage of intravenous thorium dioxide contrast, Paget’s disease of bone, chondromyxoid fibroma and previous history of radiation [8].

The histology criteria are depending on the presence of many cells with hyperchromatic nuclei, two or more chondrocytes within a lacuna and giant cartilage cells with large single or multiple nuclei with clumps of chromatin and mitotic activity [5]. Chondrosarcoma is classified by the WHO according to the degree of cellularity, nuclear size, atypia and mitotic activity as grade 1 is well differentiated (low cellularity) with abundant hyaline cartilage matrix [9], which is commonly seen in the skull base at about 90%. Grade 1 chondrosarcoma mostly affects elderly patients aged between 60 and 70 years old and rarely metastasis. Grade 2 is moderately differentiated (medium). In contrast, grade 3 chondrosarcoma is poorly differentiated (highly cellular) with a mucomyxoid matrix and mitoses [9]. Grade 3 is seen in the younger age group (between 20 and 40 years old) and has a higher rate of recurrence in about 70% of patients [10].

The 5-year survival rate of chondrosarcoma is about 70%-80%, with a good prognosis [11]. However, it is known to slow progression and can cause multiple metastases, which are usually seen in patients who have undergone multiple operations over a period. The prognosis of nasal septum chondrosarcoma depends on the location and size of the tumour and the degree of differentiation. The rates of lymph node and distant metastasis are low about 5.6%-6.7% [12].

Complete excision of the primary site with adequate surgical margin is important for a successful outcome as chondrosarcoma is considered a radio-resistant tumour. As highlighted in this case, the use of a navigation system in an expert hand, as part of the surgical adjunct will enable complete resection of the tumour endoscopically. It also spares the open surgical approach and avoids associated complications to open surgery.

According to Lee et al., postoperative radiotherapy may have some role in the treatment of chondrosarcoma as two of the cases reported with a positive margin were successfully treated with postoperative radiotherapy [11]. Adjuvant chemotherapy also plays an extremely limited role and is reserved for residual or recurrent disease and palliation [13]. Postoperative follow-up for tumour surveillance is mandatory as it allows early recognition of residual or recurrence of disease which mandates repeat biopsy and imaging for further management of the disease.

## Conclusions

Chondrosarcoma of the nasal septum is a rarely made diagnosis. Prompt diagnosis, surgical treatment and multi-disciplinary team involvement and discussion are paramount. Complete excision of the primary site with adequate margin can be curative. Long-term follow-up with endoscopic and radiological assessment is important due to the slow progression of the disease and the high rate of recurrence in the higher grade of chondrosarcoma.
